# On the Treatment of Urethritis by Cubebs and the Oleo-Resins

**Published:** 1874-06-15

**Authors:** 


### ON THE TREATMENT OF URETHRITIS BY CUBEBS AND THE OLEO-RESINS.

*From La France Medicale, of April* 11, 1874.

THERAPEUTICAL anarchy, speculation and experiment, have found a wide field of career in the treatment of urethritis. Several causes have here operated: One exists in the nature of the disease itself, which presents an almost infinite variety of form and degree of intensity, from the inflammatory and blennorrhagic disorders to the simplest catarrhal gleet; another is to be found in the anatomical seat of the disease, where every irregularity of conduct or regime is productive of fixed results. Still another lies in the influence brought to bear by the patients themselves, who, in almost every instance, desire to be treated and cured in a secret manner.

Practically, the distinction between blennorrhagia and acute urethritis, is difficult of recognition and rarely useful. Apart from their origin, where are their distinctive features to be seen? Surely a relative degree of virulence is not adequate to establish a difference. Is it not a matter of daily experience to meet with cases of simple urethritis, born of contact with a uterine catarrh, and producing, in turn, effects which are entirely similar to those of gonorrhœa? It is to be noted simply, that in the two cases there is an exclusively local contagion, exercising a reciprocal influence upon the vulvar and urethral membrane of the female, and the urethral membrane of the male. Here we find the limit of the blennorrhagic influence. In the extremely rare cases where general phenomena of syphilis succeed, there is co-existence of a separate virus, a primitive lesion of specific disease has been developed at some concealed point of the urinary conduit.

The methods of treatment heretofore employed can be classified in two great categories: 1st, external measures tending to a direct modification of the mucous membrane, and 2nd, internal remedies intended to act favorably upon the urinary canals by the intervention of the urine.

Of late there have been numerous partisans of the external method. There are always patients whose social position precludes the idea of other remedial measures, and these invariably find indefatigable allies among the interested vendors of secret and infallible injections. One variety of this method is the so-called abortive treatment, completely abandoned to-day to the consternation of our friends the urethrotomists, who find their occupation gone, so far, at least, as these cases are concerned.

To this plan the substitutive and astringent injections have largely succeeded.

Substitutive injections are, in large proportion, of empirical origin. *Substitutive irritations* having proved of immense value in oculo-palpebral catarrh, it was quite natural to presume that a similar treatment of urethral catarrh would be followed by like results. Accordingly, search was made high and low for a specific agent, which might be employed. Here unfortunately a great error was committed. In order to understand how radical the difference is between these two affections, it is only necessary to consider how the palpebral membrane is affected when blennorrhagic matter is applied to it. It is at once evident that the immense difference between simple blepharitis and purulent ophthalmia divides the two disorders, when occurring in an isolated field. Hence substitutive injections, even when employed at the outset, are rarely efficacious in urethral catarrh.

An important element in this latter affection is the frequent and irritating passage of urine over an inflamed mucous surface. The urine of the day is much more abundant than that of the night, and therefore much less irritative in every period of the urethral disorder. It is for this reason that the complete resolution of the inflammation is so often not accomplished in the later stages of the disease, and that gonorrhœa is transformed into gleet.

But therapeutic investigation once embarked in the direction of search for a specific injection, could not be stayed. Every detersive or astringent remedy was tried in turn. The sulphates of zinc and copper; the subnitrate of bismuth; the permanganate of potassa; all the mineral salts, in short, as well as the vegetable extracts, and notably tannin, were successively employed.

Tannin injections,* though greatly vaunted by Niemeyer, are rarely successful, and have no greater effect in abating the disease, when used at its commencement, than other remedies. In many instances where they seem to exert a favorable influence, they are merely palliative, and the affection, somewhat modified, persists in a sub-acute form, far beyond the limits which are prescribed by an external and internal emollient form of treatment. The esteem in which injections are held, has been sustained by the fact that internal medication with powder or extract of cubebs, copaiba, tar and opiates, has generally been employed in an insufficient and positively injurious manner. As a rule, these substances are administered in three doses daily—one at morning, another at noon, and a third at night; and here the treatment rests. Now, when given in this way, these remedies are of very slight efficacy, seeing that these doses correspond to a third merely of the urinary discharges. It results that, for one occasion where the reverse is true, there are two where the unmodified urine irritates the canal and neutralizes the beneficial effect of the medicament. And yet, in spite of these flagrant violations of common sense, this mode of treatment generally prevails in pri-

* Niemeyer injects several times during the day a solution of tannin in the ordinary "vin de pays"—or Bordeaux wine—five grains to the ounce, doubling the amount in two days if relief be not had.

vate practice and in the usage of hospitals.

In order to obtain from the powder of cubebs and the oleo-resins all the good effects of which they are capable, they must be exhibited, not three, but *ten*, *twelve*, and even *twenty* times, daily, in very acute urethritis. It should, however, be stated here, that I know of no other remedy besides powdered cubebs, which can be administered in such frequent doses with toleration by the stomach. Each dose should be nearly fifteen grains in size, and may be enveloped in a roll of wafer or other material, or, better yet, mixed with honey, so as to form an almond-sized mass. In the case of the extract, the dose is of course smaller and more easy of deglutition.

There is no denying the fact that this method of treatment is less convenient than others, but there is an offset to this in the results, which are almost immediately perceptible. I have frequently seen attacks of acute urethritis, accompanied by dysuria and chordee, amend so rapidly, that in less than forty-eight hours these phenomena disappeared; and thrice I have witnessed the same results in thirty-six hours. But, for this, it is necessary that the patient take a dose of the cubebs every hour of the day, and every hour of the night when he is awake; and that these doses be accompanied by draughts, in large quantity, of milk, to which orgeat may be added, or sweetened orange-flower water.

In these conditions the emission of urine is frequent and abundant, but in consequence of that very abundance and the soothing properties constantly communicated to it by the cubebs, the passage of the stream over the neck of the bladder and the urethra is entirely painless. But it is a mistake to suppose that at such times there is disappearance of the inflammatory condition. That condition simply does not indicate its existence by pain, but remains latent so long as treatment is regularly pursued If the latter be interrupted, the pain recurs.

Resolution is, however, gradually brought about, so that, at the expiration of eight or ten days, the number of doses may be decreased to one every two hours. Absolute rest is not imperatively demanded. Some exercise may be allowed, and here, also, the greater or less degree of painlessness of discharge, must be the measure of all excess.

Before I adopted this mode of medication, I had occasion to treat, as all have had, several acute cases by the antiphlogistic method: leeches to the perineum; sitz-baths twice daily; cataplasms, and emollient draughts; but in no one case have I seen the dysuria and chordee relieved before the eighth or tenth day. The relief of these two symptoms is much more rapid with the powdered cubebs; but resolution is not accomplished *pari passu.* The latter generally results in from fifteen to twenty days. The discharge then becomes more and more serous, and is gradually suppressed altogether between the twentieth and twenty-fifth day, without the necessity for a recurrence to injections. The average duration of treatment, then, is from twenty-two to twenty-six days for acute catarrh or blennorrhagia, and a shorter period for the milder cases.

It is now more than ten years since I have employed this method of treatment—a method, the honor of whose discovery I can in no wise claim—and I declare positively that I have obtained invariable results whenever I have had patients who desired to be promptly cured, and were willing to rigorously submit to the prescribed rules. I state this with the greater assurance, because for several years I have been enabled to experiment, on a large scale, in the military infirmaries, where attacks of urethritis almost always supply a third of the contingent diseases. This method offers an additional advantage from the circumstance that, if need be, it may be combined with antiphlogistic measures—either with astringent or absorptive injections of starch or sub-nitrate of bismuth. It is only in exceptional cases, however, that I have recourse to the latter.

Instances, however, are not rare, where a complete cure is not effected either with powdered cubebs, the balsams or injections. This generally occurs in scrofulous patients, where no external signs of the diathetic disorder are apparent, or when the urethral catarrh is complicated by an ulcerative erosion. Everyone knows that in the first instance a ferruginous preparation is indicated; and that, in the second case, a favorable termination is best secured by the passage of bougies, smeared with a more or less astringent ointment. Occasionally, however, the lesion of the membrane which produces the gleet is excessively slight, and situated so far back in the prostatic region that the bougie is inefficacious. In these conditions the capsules of the oil of turpentine, one to three being administered during the night, produce excellent results.

Gleet is always the result of vicious or insufficient treatment, or of recurrent attacks of chronic catarrh. Its frequency after the treatment by injections is such that Ricord, in his Clinical Lectures at the *Hopital du Midi* declares, with his usual imaginative and fanciful comparisons, that it is one of those complications which are forever destined to evoke the despair of the surgeon. But, with frequently repeated doses of powdered cubebs and balsams, this unfortunate issue need never, or almost never, occur; and an incalculable advance may thus be made.

Gleet is, above all else, occasioned by the special irritation of the mucous lining of the urethra, by the urine of the night, which is much more acrid than that of the day. There is, hence, a formal indication to continue the administration of the balsams *during the evening*, even when medication by day has been discontinued; and to persevere through the night with similar remedies, so long as there is apparent in the morning, the slightest trace of the characteristic moistening of the urethral surface.

The treatment recommended above, as has been already remarked, is not new. It was long since advised by a celebrated surgeon of the La Charite; and it is simply remarkable that it has attracted so little attention. During the last twelve years in which I have employed it, I have met but one physician who made use of similar measures, and, singularly enough, he supposed himself to be its discoverer. This physician, who is justly ranked among our most distinguished syphilographers, is M. Langlebert. One evening, in 1864, a friend and confrere in medicine, invited me to attend a reunion of the Medical Society of the Pantheon, where I had the good fortune to hear M. Langlebert set forth his views. He remarked that, in his opinion, powdered cubebs and the oleo-resins were of efficacy in urethritis, only as they acted by the intervention of the urine, and communicated to it medicinal properties; that, consequently, he prescribed these remedies only in doses at short and fixed intervals, so that the urine was constantly subjected to their modifying influences. This practice was, as he said, founded upon the results of his own practical experience, and there was surely no reason to doubt his statement of the fact. His surprise was therefore great, when I took the liberty to observe that the method was by no means a novel one, and could be found described at length in the fourth volume of Prof. Piorry's "Pathologie Iatrique." However, be the source of its discovery here or there, the solution of that question can neither add to, nor detract from, the excellence of its results. It is, in my opinion, not only superior to all other methods in this particular, but, also, in the point that it can always be combined with the latter in order to augment their efficiency.

NOTE BY TRANSLATOR.—Dr. Jno. W. Reigh has, recently, in the *Practitioner*, called attention to the advantage of using the bromide of potassium in blennorrhagic affections. Internally, the following formula is advised:

℞ Potassæ Carbonatis, ʒ jss.

Potassii Bromidi, ʒ jj, ad ijss.

Hyoscyami Tincturæ, f ʒ j.

Aquæ Camphoræ, f ℥ v. Mix.

A sixth of this potion is to be administered three times daily, and another sixth is to be given at night, if there be asomnia. The alkalies are added in order to correct the acidity of the urine, which the bromide of potassium frequently determines.

The following injection is advised for use every four hours:

℞ Potassii Bromidi, ʒ iij.

Glycerinæ, f ʒ v.

Aquæ Destillatæ, f ℥ v. Mix.

The bromide of potassium is said to diminish the secretion of the mucous membrane; to act directly as a sedative to the nerves distributed to it and those which preside over the organs of generation; and, lastly, to augment the quantity of the urinary secretion, thus diminishing its specific gravity, and, thereby, its capability of exciting irritation. The treatment is said to be available in all stages of the disorder, whether it be acute or chronic, without precluding the employment of accessory measures.

No greater comment could be made upon the suggestions of Dr. Ferran, detailed above, than to remark that the idea of *continuous* medication of the urethra, by the intervention of the urine, is almost entirely ignored in the text-books. I have before me at this moment the very latest work on the subject of genito-urinary diseases, just issued from the press of Messrs. Appleton & Co., under the high authority of Professors Van Buren and Keyes. The well-known posology is here described with the usual directions: "three or four times, daily, fasting;" "to be taken after eating," &c.

It should be added, however, that not a few practitioners can be found in our country, who have for years availed themselves of methods for the cure of urethral catarrh similar to, or identical with, that suggested by Ferran, to the no small profit of their patients and enhancement of their own reputations. J. N. H.

